# Generation of a Persistently Infected MDBK Cell Line with Natural Bovine Spongiform Encephalopathy (BSE)

**DOI:** 10.1371/journal.pone.0115939

**Published:** 2015-02-03

**Authors:** Dongseob Tark, Hyojin Kim, Michael H. Neale, Minjeong Kim, Hyunjoo Sohn, Yoonhee Lee, Insoo Cho, Yiseok Joo, Otto Windl

**Affiliations:** 1 Department of Animal and Plant Health Research, Animal and Plant Quarantine Agency, 175 Anyang-ro, Manan-gu, Anyang-si, Gyeonggi-do, Republic of Korea; 2 Department of Animal Disease Control and Quarantine, Animal and Plant Quarantine Agency, 175 Anyang-ro, Manan-gu, Anyang-si, Gyeonggi-do, Republic of Korea; 3 Pathology and Host Susceptibility Department, Animal Health and Veterinary Laboratories Agency, New Haw, Addlestone, KT15 3NB, United Kingdom; The Scripps Research Institute Scripps Florida, UNITED STATES

## Abstract

Bovine spongiform encephalopathy (BSE) is a zoonotic transmissible spongiform encephalopathy (TSE) thought to be caused by the same prion strain as variant Creutzfeldt-Jakob disease (vCJD). Unlike scrapie and chronic wasting disease there is no cell culture model allowing the replication of proteinase K resistant BSE (PrP^BSE^) and the further *in vitro* study of this disease. We have generated a cell line based on the Madin-Darby Bovine Kidney (MDBK) cell line over-expressing the bovine prion protein. After exposure to naturally BSE-infected bovine brain homogenate this cell line has shown to replicate and accumulate PrP^BSE^ and maintain infection up to passage 83 after initial challenge. Collectively, we demonstrate, for the first time, that the BSE agent can infect cell lines over-expressing the bovine prion protein similar to other prion diseases. These BSE infected cells will provide a useful tool to facilitate the study of potential therapeutic agents and the diagnosis of BSE.

## Introduction

Transmissible spongiform encephalopathies (TSEs) are progressive neurodegenerative disorders causing degeneration of neurons and include Creutzfeldt-Jakob disease (CJD), bovine spongiform encephalopathy (BSE), scrapie, transmissible mink encephalopathy (TME), and chronic wasting disease (CWD). In 1996 a new disease, variant CJD (vCJD) was identified with evidence suggesting that vCJD and BSE are caused by the same prion strain. vCJD is most likely caused from consumption of contaminated beef or beef by-products [1]. The causative agent of TSEs most likely is an infectious protein (PrP^Sc^) which unlike bacteria and viruses, does not contain any nucleic acid to propagate itself. PrP^Sc^ is generated from a normal host-encoded cellular prion protein (PrP^C^) during disease and is conformational different to the normal cellular protein [2]. These conformational differences result in an increased resistance to degradation allowing for detection of the disease associated PrP^Sc^.

The development of cell lines for a specific prion disease may be advantageous for a variety of studies, for example, screening of anti-prion substances, formation and inhibition of pathogenic prions [3–6]. However, the availability of cells susceptible for TSE infection is still very limited. The majority of susceptible cells are mouse-derived [7,8]. In addition, propagation of chronic wasting disease (CWD) has been successfully achieved in a mule deer-derived fibroblast-like cell line [9] and rabbit RK13 cells expressing elk PrP and the HIV-1 GAG precursor protein (RKE-Gag) [10]. However, no cells susceptible to infection with natural BSE from cattle have been established. To date, BSE related research relies heavily on the use of mice or transgenic mice expressing animal species-specific PrP^C^ [11,12], or on large animal studies [13]. There is a strong requirement for replacing the animal models with *in vitro* systems using cell lines susceptible to BSE infection, to reduce the time and cost of such studies. Such systems will significantly facilitate the diagnosis of BSE as well as the study of potential therapeutic agents and disease pathogenesis.

In this study, we report for the first time a cell line which is persistently infected with BSE utilizing Madin-Darby Bovine Kidney (MDBK) cells over-expressing bovine PrP established using a lentiviral expression system. These results provide evidence that PrP^BSE^ is able to replicate persistently in an *in vitro* cell culture.

## Materials and Methods

### Prion protein genes (PRNPs) and cloning

Primer sequences were designed against the bovine PRNP gene (GenBank: AJ298878) and, for cloning, *Not*I and *Xho*I restriction enzyme sites were added to a forward primer (5'-AGCGGCCGCGCCACCATGGTGAAAAGCCACATAGG-3') and a reverse primer (5'-CGGCTCGAGCTATCCTACTATGAGAAAAATGA-3'), respectively. Using the above primer pair, the complete PRNP coding region from brain tissues of cattle (Korean native cattle; Hanwoo) which were collected from abattoir (Hyup Sin Food Co. Ltd., Korea) was amplified by PCR as follows: initial denaturing at 94°C for 5 min, followed by 30 cycles of 94°C for 30 seconds, 60°C for 30 seconds and 72°C for 90 seconds, and a final elongation stage at 72°C for 15 min. The amplicons were cloned into pGEM-T Easy vector (Promega) and DNA sequencing of the target genes were performed (Macrogen Inc., Korea).

### Infectious recombinant lentiviruses

Infectious recombinant lentiviruses were produced following published methods [14,15] by Macrogen Inc.(Seoul, Korea). Briefly, the cloned PRNPs were inserted into pLEX MCS transfer vector (Thermo Scientific Open Biosystems) using restriction enzymes *Not* I and *Xho* I (New England Biolabs). This cloned transfer vector was then mixed with a VSV-G expression vector and a gag-pol expression vector in a relative molar ratio of 1:1:1, and co-transfected into 293T cells using lipofectamine Plus (Invitrogen, USA). The cell culture supernatant containing recombinant virus was recovered 48 hours after transfection, and filtered using a membrane filter with a pore size of 0.45 μm (Nalgene, USA), and stored immediately at −70°C. A titer value of the infectious recombinant virus was indirectly measured in HeLa cell, using fluorescent microscopy, to detect GFP expression in the transduced cell which has transfected with only pLEX vector containing GFP gene instead of bovine PRNP.

### Cell and transduced cell lines

MDBK cell was obtained from the American Type Cell Collection (ATCC). Cells were grown in completed medium (Dulbecco’s modified Eagle’s medium/F12 supplemented with 10% fetal bovine serum, antibiotics (penicillin and streptomycin), non-essential amino acid, and L-glutamine). To determine puromcyin concentration for selection of transduced cells, cell lines were treated with 0 to 10 μg/ml of puromycin and cultured for 3 ~ 4 days and observed for cell death. The optimal concentration was determined to be in the range of 1.5 to 2.5 μg/mL and used for selecting the transduced cell.

The transduced cell lines were produced as follows: Day 1 before recombinant lentiviral infection, cell lines were plated on a 12-well plate for culture and allowed to grow to 60 to 70% confluence on the inoculation day. After removing the culture supernatant, 0.5 mL of infectious recombinant lentivirus was inoculated and 8 ㎍/mL of Polybrene (Hexodimethrine bromide, Sigma H9268) was added, followed by gently shaking the mixture. After incubating overnight (for 15 to 16 hours) at 37°C under 5% CO_2_ atmosphere, the inoculum was replaced by fresh completed medium and, on the following day, puromycin was added to the culture medium and transduced cells were selected while continuously culturing for 7 to 14 days.

### Immunofluorescence assay

The transduced cells or the normal cells were cultured in chamber slide (Nunc, Lab_TeK chamber slide) and fixed with 4% paraformaldehyde (PFA, Sigma P6148) for 30 minutes and dried in order to determine whether or not prion protein was expressed on the cell membranes. A primary antibody, 6H4 (Prionics, 1 mg/mL, 1:100) was added to the fixed cells, and incubated for 1 hour at room temperature. After washing the cells 3 times using phosphate buffered saline (PBS), anti-mouse IgG FITC conjugate (KPL; Kirkegaard & Perry Laboratories, 0.5 mg/mL, 1:200) was added and incubated for 1 hour at room temperature. After washing the cells 6 times using PBS they were mounted and observed on a fluorescent microscope (Olympus, Tokyo, Japan).

### Immunoblotting

Cultured cells were washed once using PBS and adherent cells collected from the culture flask in PBS using a scraper and transferred to a centrifuge tube. After centrifugation at 1,200 rpm for 5 minutes to pellet the cells, the supernatant was discarded and the pellet was dispersed by gently tapping the tube. The cells were lysed by adding lysis buffer (0.5% Triton X-100, 0.5% sodium deoxycholate, 10 mM Tris-HCl [pH7.5], 150 mM NaCl, 5 mM EDTA) to the suspension, and incubated on ice for 30 minutes. After this incubation, the product was subjected to refrigerated centrifugation at 1,200 rpm for 5 minutes and the supernatant was transferred into a new tube.

For PrP^C^ analysis, using Dynabeads (Dynabeads M-280 Sheep anti-mouse IgG) coupled to 6H4 (1.5 μg of 6H4 per 30 μL beads suspension), the supernatant was subjected to a semi-purification according to the manufacture instructions. For PrP^Sc^ analysis, the supernatant containing ∼ 0.5 − 1 mg of total protein was incubated with 20 μg/mL proteinase K for 20 min at 37°C prior to addition of 1 mM 4-(2-Aminoethyl)-benzenesulfonyl fluoreide (Roche) and incubation for 5 minutes in ice. The proteinase K treated proteins were centrifuged at 20,000 x g for 2 hours and the pelleted protein was resuspended in 25 μL 1 x sample loading buffer (Invitrogen). For removing N-linked glycosylation from PrP^Sc^ in cells before PK digestion, PNGase F was used according to the manufacturer’s recommendation (New England Biolabs). US/FluorchemQ analysis (Alpha Innotech, USA) was used for estimating migration of ungycosylated PrP after treatment of PNGase F.

The electrophoresis was conducted at 200 V for 35 minutes using a pre-casted NuPAGE 12% Bis-Tris gel (Invitrogen) and proteins were transferred to a polyvinylidene difluoride (PVDF) membrane by an electrical device at 150 V for 1 hour. After transfer the PVDF membrane was blocked using a blocking solution comprising 0.02% I-block (Tropix) dissolved in a tris-buffered saline (TBS), the membrane was incubated with the 6H4 monoclonal antibody (1 mg/mL, 1:3,000) or rabbit anti-PrP polyAb S1 (produced by the Animal and Plant Quarantine Agency, Korea) at room temperature for 1 hour. The membrane was washed 3 times using TBST (which comprises 0.05% (v/v) Tween 20 in TBS) and reacted with goat anti-mouse IgG (or anti-rabbit IgG) alkaline phosphatase-conjugated secondary antibody (KPL, 1 mg/mL, 1:3,000) at room temperature for 1 hour. Finally, the membrane was washed 3 times using TBST for 5 minutes and expression of prion protein was visualized using CDP star (Applied Biosystem). Chemiluminescence signals were analyzed using the LAS 4000 imaging system (Fuji, Japan).

### ELISA and scrapie cell assay

To determine whether PrP^Sc^ infection was established in the transduced cell, a bovine brain homogenate from classical BSE infected cattle (supplied by TSE Archive, Animal Health and Veterinary Laboratories Agency UK) was prepared and used to challenge these cells according to the following procedure. First, the BSE brain homogenate was diluted in DMEM supplemented with 100 ng/mL of phorbol 12-myristrate 13-acetate (PMA, Sigma P8139), 10% fetal bovine serum (Gibco-BRL 16000–044), antibiotics (Gibco-BRL 15140–155), non-essential amino acids (Gibco-BRL 11140–050) to a final concentration of 0.25% and 100 μL of the diluted homogenate was inoculated onto a 96-well tissue culture plate containing 60 to 70% confluent cells. Once confluent, cells were serially transferred to a 24-well tissue culture plate, a 25 cm^2^ flask and a 75 cm^2^ flask at 4 to 6 day intervals.

BSE infection of the cultured cells in the 75 cm^2^ flask was analyzed by ELISA (IDEXX HerdCheck) according to the manufacturer’s instructions. The transduced cells were subjected to limiting dilution using a 96-well plate after confirmation of infection by ELISA. The limiting dilution was performed by plating out an average of 0.3 cells per well of a 96-well plate. The individual clones were screened using the published protocol of the standard scrapie cell assay (SCA) [16] modified and performed as below. The single cloned cells were propagated until confluent. About 25,000 cells per well were suspended in DMEM containing 10% fetal bovine serum and transferred into each well of 96-well enzyme linked immune-spot assay (ELISPOT) plates (MultiScreen-IP filter plate; Millipore). Plates were dried and proteinase K (PK) treated (1 μg/mL in lysis buffer), followed by protease inhibition with phenylmethylsulfonyl fluoride (PMSF; sigma) and denaturation with Tris-guanidinium thiocyanate (GSCN). The denaturated PrP^Sc^ proteins were detected with the primary antibody, 6H4 (1 mg/mL, 1:3,000) and secondary antibody, alkaline phosphatase–conjugated anti-mouse IgG (1 mg/mL, 1:3,000, KPL) and detected with alkaline phosphatase conjugate substrate, BCIP/NBT (KPL). The immunostained spots were visualized with CTL ELISPOT equipment (Cellular Technology, Ltd, Shaker Heights, OH)

## Results

### Production of infectious recombinant lentiviruses and transduced MDBK

In order to produce infectious recombinant lentivirus for transducing the MDBK cell line and expressing recombinant bovine prion protein, bovine prion protein gene (PRNP) was amplified by PCR. The amplified bovine PRNP was compared with accession numbers AJ298878 (100%), and EU224471 (99.87%) registered in GenBank, it contained 6 octarepeats. The infectious recombinant lentivrus was produced using the amplified bovine PRNP as described in materials and methods. The measured titer of the lentivirus was more than 10^6^ transduction unit (TU) as determined by fluorescent antibody (FA) test (data not shown).

**Figure 1:**
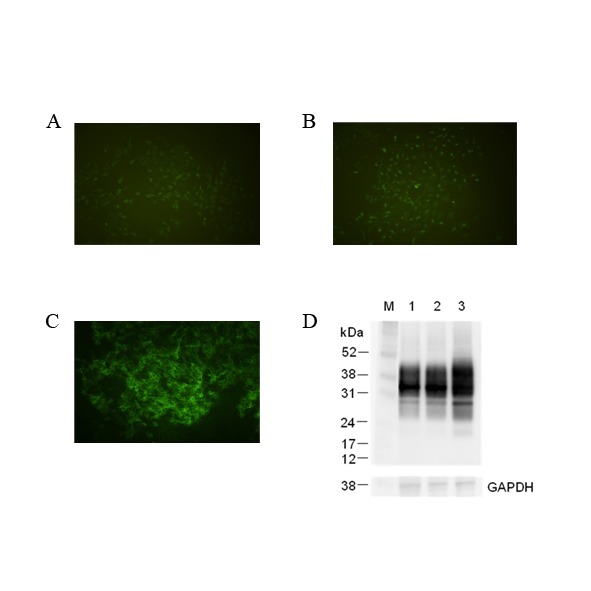
Immunofluorescence and Immunoblotting detection of bovine cellular prion protein (PrPC) in transduced/non-transduced Madin-Darby bovine kidney (MDBK; ATCC CCL-22) with infectious recombinant lentivirus. Detection of cell surface PrP^C^ in non-transduced MDBK (A), transduced MDBK with only pLEX vector (B) and MDBK C1–2F with bovine PRNP inserted into pLEX vector (C) were assayed by immunofluorescence using 4% paraformaldehyde fixation and anti-PrP mAb 6H4. (D) This photo shows an immunoblot of non-transduced MDBK (lane 1) and transduced MDBKs with infectious recombinant virus which had pLEX vector without and with bovine PRNP (lane 2, 3). Housekeeping protein, GAPDH (glyceraldehydes-3-phosphate dehydrogenase) was used as a control of comparative protein concentration. The positions of molecular marker proteins (M) are presented in kilodaltons.

The transduced MDBK cell line was produced using recombinant lentivirus containing bovine PRNP and puromycin resistance to select transduced cell clones. In Fig. 1, we compared to the non-transduced MDBK control (A), the transduced MDBK with only pLEX vector (B) and MDBK C1–2F with bovine PRNP inserted into pLEX vector (C) for the expression of prion protein on their cell surface. MDBK C1–2F cells showed that PrP^C^ was highly expressed on the cell surface compared with the controls (MDBK cells express moderate levels of intrinsic PrP^C^). By Western blot (WB), the prion protein expression levels between the three types of cells was determined by comparison with the house-keeping protein GAPDH. The transduced MDBK cell, MDBK C1–2F cell, exhibits the highest expression level of prion protein (Fig. 1D, lane 3).

### Production of PrP^BSE^ infected cell line

**Figure 2:**
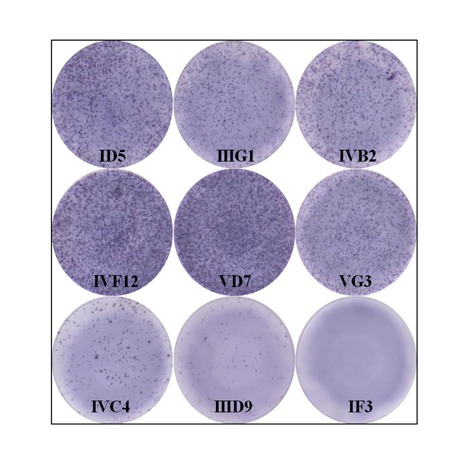
Confirmation and cloning of PrP^BSE^-infected cells. The presence of proteinase K resistant PrP^BSE^ in 96 well elispot plates was confirmed by using scrapie cell assay (SCA). The photograph showed severe (ID5, IIIG1, IVB2, IVF12, VD7 and VG3), moderate (IVC4), weak (IIID9) and non-infected (IF3) wells. These data shown are representative of results from ELISA and Western blot.

In order to determine whether PrP^Sc^ infection could occur in the bovine PrP transduced cells, a 0.25% (w/v) bovine brain homogenate from naturally BSE infected cattle was used to challenge the transduced cells. BSE infection was firstly identified by ELISA performed on the challenged cultured cells from a 75 cm^2^ flask (data not shown). The infected cells were subjected to limiting dilution using 96-well plates to isolate pure infected clones. Isolated clones were analysed using the scrapie cell assay [16]. Six wells (ID5, IIIG1, IVB2, IVF12, VD7 and VG3) out of 5 plates (480 wells plated) showed strong positive responses and two showed weak positive responses (IVC4 and IIID9), see Fig. 2. In addition, the optical densities (mean ± standard deviation, n = 7) of IVF12 and VD7 were found to be 3.118 ± 0.026 and 3.151 ± 0.007 respectively. These values when compared with the value of 0.05 ± 0.004 of MDBK C1–2F control cells confirmed the results of the SCA and indicated that the cells are infected with naturally occurring BSE.

### Confirmation of persistent PrP^BSE^ infected cell (M2B cell)

**Figure 3:**
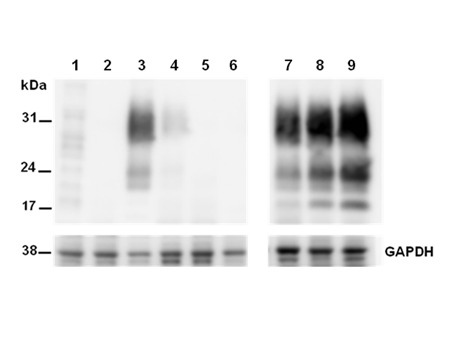
Comparison of persistently infective abilities on non-cloned and cloned cells successively passaged. Lane 1 and 2, normal MDBK C1–2F (without and with PK treatments); Lane 3 to 6, uncloned BSE-infected MDBK C1–2F (passage level; p11, p17, p24, and p27); Lane 7 and 9, cloned BSE-infected MDBK C1–2F (passage level; p15, p19, and p26). Molecular mass marker in kilodaltons (kDa) is shown on the left. Housekeeping protein, GAPDH (glyceraldehydes-3-phosphate dehydrogenase) was used as a control of comparative protein concentration. The result shown is representative of three independent experiments.

**Figure 4:**
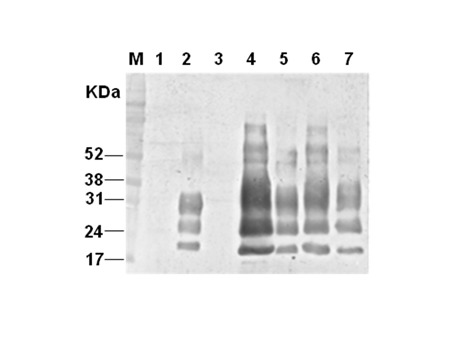
Immunoblotting detection of the persistent PrP^BSE^-infected cell according to serial passages. The lysates of brain homogenates (lane 1; BSE-noninfected bovine brain, lane 2; BSE-infected bovine brain-original BSE source) and MDBK cells expressing normal bovine prion protein by using infectious recombinant lentivirus (lane 3: transduced MDBK–MBDK C1–2F–passage(p) 7; lane 4: BSE-infected transduced MDBK (M2B)–p17; lane 5: M2B-p48; lane 6: M2B-p70 and lane 7: M2B-p83) were treated with proteinase K (PK) for detection of PrP^BSE^ in transduced MDBK which was sequentially passaged after inoculating BSE-infected bovine brain homogenate. Molecular mass marker (M) in kilodaltons (kDa) is shown on the left. The result shown is representative of multiple independent experiments.

**Figure 5:**
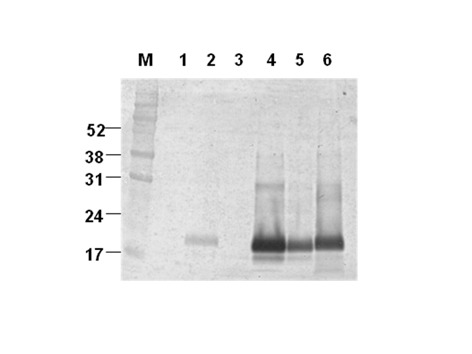
Comparison of the mobilities of PK-resistant PrP derived from BSE-infected bovine brain (original cell inoculate) and M2B cell. Mobility phases of nonglycosylated PrP^BSE^ from the non-infected bovine brain (lane 1), BSE-infected bovine brain (lane 2), non-infected transduced MDBK (MDBK C1–2F, passage (p) 7: lane 3), and persistently PK-resistant PrP^BSE^ infected M2B cells (p17: lane 4; p48: lane 5 and p70: lane 6) in Western blotting after sequential treatments with PK and PNGase F. Molecular mass marker (M) in kilodaltons (kDa) is shown on the left.

To confirm whether these cells were persistently infected, one clone, VD7 (see Fig. 2) was passaged further and cloned by limiting dilution adjusting the number of cells to approximately 0.3 cells per well in 96-well plate. As shown in Fig. 3, proteinase K resistant PrP^Sc^ in the BSE infected uncloned cells disappeared before undergoing 20 passages (lane 3 to 6 in Fig. 3). However, the cloned infected cells, M2B (stand by MDBK with 2-times cloning after challenge of BSE infected bovine brain homogenate) exhibited persistent infection, proteinase K resistant PrP^Sc^ even after 26 passages by continuous subculture (lane 7 to 9 in Fig. 3). In contrast the unchallenged control cells have no proteinase K resistant prion protein band (lane 2 of Fig. 3), since normal prion protein in these cells was completely degraded by proteinase K treatment. The infected cells maintained their infection as evidenced by a proteinase K resistant PrP^Sc^ signal and is consistently seen throughout subsequent passages up to passage 83 (Fig. 4 lanes 4–7). In comparison to the nonglycosylated PrP^BSE^ of the original BSE inoculum (Fig. 4, lane 2 and Fig. 5 lane 2 after PNGase F deglycosylation), those of the cell line derived PrP^BSE^ have a lower molecular weight (about 1.3 kDa) as detected by WB (Figs. 4 and 5).

## Discussion

Until now, BSE susceptible cell lines have not been developed. Although Heining *et*.*al*. [17] recently have shown that PrP^res^ could be detected in the PrP^0/0^ Chimp1 cell clone after incubation with BSE infected cow brain homogenate, this cell line has not been established as a persistently infected cell line against natural BSE infected bovine materials. Various cell lines have been developed which are susceptible to infection mainly with murine-adapted or ovine scrapie-infected materials and also recently two cell lines have been produced allowing infection with chronic wasting disease (CWD)[10,18–21]. No reliable cell model exists for human prion diseases including variant Creutzfeldt-Jakob disease (vCJD) although the human neuroblastoma cell line SH-SY5Y was shown to be permissive to human prion infection [22].

In this study, we have produced a persistently BSE-infected cell line, M2B, which overexpresses bovine prion protein in MDBK cells using a lentiviral expression system [14,15], and infected these cells with bovine brain homogenate from cattle infected with classical BSE. Prior to establishing this cell line we attempted to use primary bovine brain cells, especially neuronal cells however these cells were not easily immortalized using simian virus 40 large T antigen [23] or successfully passaged after infection (data not shown). Non-neuronal cells have been reported to be permissive to infection with prion diseases [9,21,24,25]. Vorberg *et*. *al*. [25] demonstrated that the mouse fibroblast cell lines NIH/3T3 and L929 are permissive to infection with mouse-adopted scrapie in spite of expressing low level of cellular mouse prion protein. Here we have used MDBK to generate a BSE susceptible cell line, although PrP^C^ expression level of kidney is lower than any other tissue [26] and, to our knowledge there are no reports of PrP^Sc^ detection from bovine kidney. Ryder *et al*. and Hadlow *et al*. [27,28] found that PrP^Sc^ has been detected in the kidney of cats with feline spongiform encephalopathy (FSE) and experimental mink with transmissible mink encephalopathy (TME). Additionally cell lines based on the rabbit kidney cell line RK13 have been shown to be susceptible to a range of prion disease [29,30]. On the basis of above reports, we over-expressed bovine prion protein in MDBK using a lentiviral expression system to increase expression level of prion protein to potentially enhance susceptibility to prion infection [14,15]. As shown in Fig. 1, the recombinant prion protein is well expressed on the cell surface of bovine PRNP transfected cell compared to control cells but all cells express prion protein in cell lysates as shown by immunoblot. In our infection test, the control cells, non-transduced and empty vector transfected only, did not propagate prion infection, whereas the bovine PRNP transduced cell lines were able to be infected.

Recently studies were reported that the plasma membrane is the initial or potential site of prion conversion [31,32], so this cell surface-expressed pattern may be considered as an important factor in establishing the persistently BSE-infected M2B cell line in this study.

Because of the low infection rate in infected cell clones, we added PMA to the culture media which activates protein kinase C (PKC) and increases expression levels of PrP^C^ in cells [33,34] potentially increasing the PrP^Sc^ detection rates. Furthermore, the highly sensitive cell-based infectivity assay, the scrapie cell assay (SCA), was employed and this assay can potentially detected single infected cells [16]. In this study, we obtained 6 strongly infected cell clones from five 96-well plates using SCA. On the basis of our result, the number of infected cells is very limited as previously reported [9,35], especially in uncloned cells where infection was lost upon continuous passaging compared with cloned cells (Fig. 3). Therefore in order to obtain persistently infected cells it is required to clone the cells in order to acheive cell lines with a high proportion of infected cells. These results could be explained (1) by the lack of stability of cells during prolonged propagation [16] or (2) by a kinetic argument, i.e. persistent infection of cell is only achieved when the rate of prion synthesis exceeds degradation [36].

When PrP^Res^ from M2B cells was compared to that of the original BSE inoculum, nonglycosylated PrP^Res^ derived from cells had a slightly smaller molecular weight (Fig. 5). This result is similar to the pattern of unglycosylated PrP^Res^ from SMB cell infected with the Chandler scrapie [37] and from Rov and MovS cells infected with the 127S scrapie [38]. The former reports suggested that the difference of migration pattern in WB might be resulted from conformational differences in between cell and host brain [37,39] or through differences in the composition of the GPI anchor[40,41]. More recently Dron et al. shows that PrP^Sc^ endogenous truncation may be greater in cell lines than in brain[38].

Additionally, we examined whether PrP^BSE^ from M2B cell transmits to conventional VM mice. PrP^BSE^ from M2B was successfully infected to VM mouse and its phenotypes were consistent with those of PrP^BSE^ classical BSE (data not shown). Furthermore, we have tried to apply the lentiviral system to produce other TSE agent-infected cells. A persistently CWD infected cell line which was inoculated with naturally CWD-infected elk brain homogenate has been successfully developed in elk PrP expressing cells that were established using this lentiviral expression system [39].

In conclusion, we have demonstrated the generation of a persistently BSE-infected cell line which over-expresses bovine PrP based on the MDBK cell line. This successful achievement of generating BSE infected cells provides a methodology to potentially produce human prion infected cell models, including for vCJD, using a lentiviral expression system and also provides a research tool for research on the diagnosis of BSE and other related prion diseases as well as the study of potential therapeutic agents.

## References

[1] Will RG, Ironside JW, Zeidler M, Cousens SN, Estibeiro K, et al (1996) A new variant of Creutzfeldt-Jakob disease in the UK. Lancet 347: 921–925. 8598754 10.1016/s0140-6736(96)91412-9

[2] Prusiner SB (1998) Prions. Proc Natl Acad Sci U S A 95: 13363–13383. 9811807 10.1073/pnas.95.23.13363 PMC33918

[3] Féraudet C, Morel N, Simon S, Volland H, Frobert Y, et al (2005) Screening of 145 Anti-PrP Monoclonal Antibodies for Their Capacity to Inhibit PrPSc Replication in Infected Cells. Journal of Biological Chemistry 280: 11247–11258. 15618225 10.1074/jbc.M407006200

[4] Pankiewicz J, Prelli F, Sy M-S, Kascsak RJ, Kascsak RB, et al (2006) Clearance and prevention of prion infection in cell culture by anti-PrP antibodies. European Journal of Neuroscience 23: 2635–2647. 16817866 10.1111/j.1460-9568.2006.04805.x PMC1779824

[5] Iwamaru Y, Shimizu Y, Imamura M, Murayama Y, Endo R, et al (2008) Lactoferrin induces cell surface retention of prion protein and inhibits prion accumulation. Journal of Neurochemistry 107: 636–646. 10.1111/j.1471-4159.2008.05628.x 18717818

[6] Kocisko DA, Baron GS, Rubenstein R, Chen J, Kuizon S, et al (2003) New Inhibitors of Scrapie-Associated Prion Protein Formation in a Library of 2,000 Drugs and Natural Products. J Virol 77: 10288–10294. 12970413 10.1128/JVI.77.19.10288-10294.2003 PMC228499

[7] Iwamaru Y, Takenouchi T, Ogihara K, Hoshino M, Takata M, et al (2007) Microglial Cell Line Established from Prion Protein-Overexpressing Mice Is Susceptible to Various Murine Prion Strains. J Virol 81: 1524–1527. 17121794 10.1128/JVI.01379-06 PMC1797525

[8] Butler DA, Scott MR, Bockman JM, Borchelt DR, Taraboulos A, et al (1988) Scrapie-infected murine neuroblastoma cells produce protease-resistant prion proteins. J Virol 62: 1558–1564. 3282080 10.1128/jvi.62.5.1558-1564.1988 PMC253182

[9] Raymond GJ, Olsen EA, Lee KS, Raymond LD, Bryant PK, et al (2006) Inhibition of Protease-Resistant Prion Protein Formation in a Transformed Deer Cell Line Infected with Chronic Wasting Disease. J Virol 80: 596–604. 16378962 10.1128/JVI.80.2.596-604.2006 PMC1346862

[10] Bian J, Napier D, Khaychuck V, Angers R, Graham C, et al (2010) Cell-Based Quantification of Chronic Wasting Disease Prions. J Virol 84: 8322–8326. 10.1128/JVI.00633-10 20519392 PMC2916541

[11] Baron T, Vulin J, Biacabe A-G, Lakhdar L, Verchere J, et al (2011) Emergence of Classical BSE Strain Properties during Serial Passages of H-BSE in Wild-Type Mice. PLoS ONE 6: e15839 10.1371/journal.pone.0015839 21264286 PMC3021503

[12] Bruce M, Chree A, McConnell I, Foster J, Pearson G, et al (1994) Transmission of Bovine Spongiform Encephalopathy and Scrapie to Mice: Strain Variation and the Species Barrier. Philosophical Transactions: Biological Sciences 343: 405–411. 7913758 10.1098/rstb.1994.0036

[13] Stack MJ, Moore SJ, Vidal-Diez A, Arnold ME, Jones EM, et al (2011) Experimental Bovine Spongiform Encephalopathy: Detection of PrPSc in the Small Intestine Relative to Exposure Dose and Age. Journal of Comparative Pathology 145: 289–301. 10.1016/j.jcpa.2011.01.010 21388635

[14] Antonia F, Laurie EA, Silvia B, Massimo G, Naldini L (2000) Gene transfer by lentiviral vectors is limited by nuclear translocation and rescued by HIV-1 pol sequences. Nature Genetics 25: 217–222 10835641 10.1038/76095

[15] Dull T, Zufferey R, Kelly M, Mandel RJ, Nguyen M, et al (1998) A Third-Generation Lentivirus Vector with a Conditional Packaging System. J Virol 72: 8463–8471. 9765382 10.1128/jvi.72.11.8463-8471.1998 PMC110254

[16] Klöhn P-C, Stoltze L, Flechsig E, Enari M, Weissmann C (2003) A quantitative, highly sensitive cell-based infectivity assay for mouse scrapie prions. Proceedings of the National Academy of Sciences 100: 11666–11671. 14504404 10.1073/pnas.1834432100 PMC208815

[17] Heinig L, Mueller DA, Ramljak S, Holznagel E, Stuke AW (2010) Inducible expression of chimpanzee prion protein (PrP) in murine PrP knock-out cells. Protein Expression and Purification 70: 129–136. 10.1016/j.pep.2009.09.015 19796688

[18] Vilette D (2008) Cell models of prion infection. Vet Res 39: 10 18073097 10.1051/vetres:2007049

[19] Nishida N, Harris DA, Vilette D, Laude H, Frobert Y, et al (2000) Successful Transmission of Three Mouse-Adapted Scrapie Strains to Murine Neuroblastoma Cell Lines Overexpressing Wild-Type Mouse Prion Protein. Journal of Virology 74: 320–325. 10590120 10.1128/jvi.74.1.320-325.2000 PMC111542

[20] Iwamaru Y, Takenouchi T, Ogihara K, Hoshino M, Takata M, et al (2007) Microglial Cell Line Established from Prion Protein-Overexpressing Mice Is Susceptible to Various Murine Prion Strains. Journal of Virology 81: 1524–1527. 17121794 10.1128/JVI.01379-06 PMC1797525

[21] Courageot M-P, Daude N, Nonno R, Paquet S, Di Bari MA, et al (2008) A cell line infectible by prion strains from different species. Journal of General Virology 89: 341–347. 18089759 10.1099/vir.0.83344-0

[22] Ladogana A, Liu Q, Geng Xi Y, Pocchiari M (1995) Proteinase-resistant protein in human neuroblastoma cells infected with brain material from Creutzfeldt-Jakob patient. The Lancet 345: 594–595. 10.1016/s0140-6736(95)90508-1 7776812

[23] Jha KK, Banga S, Palejwala V, Ozer HL (1998) SV40-Mediated Immortalization. Experimental Cell Research 245: 1–7. 9828095 10.1006/excr.1998.4272

[24] Vilette D, Andreoletti O, Archer F, Madelaine MF, Vilotte JL, et al (2001) Ex vivo propagation of infectious sheep scrapie agent in heterologous epithelial cells expressing ovine prion protein. Proceedings of the National Academy of Sciences 98: 4055–4059. 11259656 10.1073/pnas.061337998 PMC31178

[25] Vorberg I, Raines A, Story B, Priola SA (2004) Susceptibility of Common Fibroblast Cell Lines to Transmissible Spongiform Encephalopathy Agents. Journal of Infectious Diseases 189: 431–439. 14745700 10.1086/381166

[26] Peralta OA, Eyestone WH (2009) Quantitative and qualitative analysis of cellular prion protein (PrPC) expression in bovine somatic tissues. Prion 3: 161–170. 19806026 10.4161/pri.3.3.9772 PMC2802781

[27] Ryder SJ, Wells GAH, Bradshaw JM, Pearson GR (2001) Inconsistent detection of PrP in extraneural tissues of cats with feline spongiform encephalopathy. Veterinary Record 148: 437–441. 11338713 10.1136/vr.148.14.437

[28] Hadlow WJ, Race RE, Kennedy RC (1987) Temporal distribution of transmissible mink encephalopathy virus in mink inoculated subcutaneously. J Virol 61: 3235–3240. 2957510 10.1128/jvi.61.10.3235-3240.1987 PMC255903

[29] Vilette D, Andreoletti O, Archer F, Madelaine MF, Vilotte JL, et al (2001) Ex vivo propagation of infectious sheep scrapie agent in heterologous epithelial cells expressing ovine prion protein. Proc Natl Acad Sci U S A 98: 4055–4059. 11259656 10.1073/pnas.061337998 PMC31178

[30] Courageot MP, Daude N, Nonno R, Paquet S, Di Bari MA, et al (2008) A cell line infectible by prion strains from different species. J Gen Virol 89: 341–347. 18089759 10.1099/vir.0.83344-0

[31] Paquet S, Daude N, Courageot M-P, Chapuis J, Laude H, et al (2007) PrPc Does Not Mediate Internalization of PrPSc but Is Required at an Early Stage for De Novo Prion Infection of Rov Cells. J Virol 81: 10786–10791. 17626095 10.1128/JVI.01137-07 PMC2045457

[32] Goold R, Rabbanian S, Sutton L, Andre R, Arora P, et al (2011) Rapid cell-surface prion protein conversion revealed using a novel cell system. Nat Commun 2: 281 10.1038/ncomms1282 21505437 PMC3104518

[33] Liu T, Li R, Pan T, Liu D, Petersen RB, et al (2002) Intercellular Transfer of the Cellular Prion Protein. Journal of Biological Chemistry 277: 47671–47678. 12359724 10.1074/jbc.M207458200

[34] Manuelidis L, Yu Z-X, Barquero N, Mullins B (2007) Cells infected with scrapie and Creutzfeldt–Jakob disease agents produce intracellular 25-nm virus-like particles. Proceedings of the National Academy of Sciences 104: 1965–1970. 17267596 10.1073/pnas.0610999104 PMC1794316

[35] Race RE, Fadness LH, Chesebro B (1987) Characterization of Scrapie Infection in Mouse Neuroblastoma Cells. Journal of General Virology 68: 1391–1399. 3106566 10.1099/0022-1317-68-5-1391

[36] Weissmann C (2004) The state of the prion. Nat Rev Micro 2: 861–871. 10.1038/nrmicro1025 15494743

[37] Birkett CR, Hennion RM, Bembridge DA, Clarke MC, Chree A, et al (2001) Scrapie strains maintain biological phenotypes on propagation in a cell line in culture. EMBO J 20: 3351–3358. 11432823 10.1093/emboj/20.13.3351 PMC125505

[38] Dron M, Moudjou M, Chapuis J, Salamat MKF, Bernard J, et al (2010) Endogenous Proteolytic Cleavage of Disease-associated Prion Protein to Produce C2 Fragments Is Strongly Cell- and Tissue-dependent. Journal of Biological Chemistry 285: 10252–10264. 10.1074/jbc.M109.083857 20154089 PMC2856230

[39] Kim H-J, Tark D-S, Lee Y-H, Kim M-J, Lee W-Y, et al (2012) Establishment of a Cell Line Persistently Infected with Chronic Wasting Disease Prions. Journal of Veterinary Medical Science 74: 1377–1380. 22673102 10.1292/jvms.12-0061

[40] Jiménez-Huete A, Lievens PMJ, Vidal R, Piccardo P, Ghetti B, et al (1998) Endogenous Proteolytic Cleavage of Normal and Disease-Associated Isoforms of the Human Prion Protein in Neural and Non-Neural Tissues. The American Journal of Pathology 153: 1561–1572. 9811348 10.1016/S0002-9440(10)65744-6 PMC1853409

[41] Arima K, Nishida N, Sakaguchi S, Shigematsu K, Atarashi R, et al (2005) Biological and Biochemical Characteristics of Prion Strains Conserved in Persistently Infected Cell Cultures. Journal of Virology 79: 7104–7112. 15890950 10.1128/JVI.79.11.7104-7112.2005 PMC1112108

